# Supplementary material for: Acute psychological and physiological benefits of exercising with virtual reality
Source: PLoS One. 2024 Dec 18;19(12):e0314331. doi: 10.1371/journal.pone.0314331 (PMC11654962; doi:10.1371/journal.pone.0314331)
Supplement: Attachment — Submitted filename: Response to Reviewers.docx [file pone.0314331.s002.docx]

An unmarked version of your revised paper without tracked changes. You should upload this as a separate file labelled 'Manuscript'.

**Journal requirements:**

**When submitting your revision, we need you to address these additional requirements.**

1. Please ensure that your manuscript meets PLOS ONE's style requirements, including those for file naming. The PLOS ONE style templates can be found at

[https://journals.plos.org/plosone/s/file?id=wjVg/PLOSOne_formatting_sample_main_body.pdf](https://eur03.safelinks.protection.outlook.com/?url=https%3A%2F%2Fjournals.plos.org%2Fplosone%2Fs%2Ffile%3Fid%3DwjVg%2FPLOSOne_formatting_sample_main_body.pdf&data=05%7C02%7Coliver.runswick%40kcl.ac.uk%7Ca38d90e72c0b4149f6a308dcb4ff158c%7C8370cf1416f34c16b83c724071654356%7C0%7C0%7C638584256008990120%7CUnknown%7CTWFpbGZsb3d8eyJWIjoiMC4wLjAwMDAiLCJQIjoiV2luMzIiLCJBTiI6Ik1haWwiLCJXVCI6Mn0%3D%7C0%7C%7C%7C&sdata=bhMQ3Pk4Mjj%2Bxl0YTv6nYhiCan5YEssseOagoWiK6HI%3D&reserved=0) and

[https://journals.plos.org/plosone/s/file?id=ba62/PLOSOne_formatting_sample_title_authors_affiliations.pdf](https://eur03.safelinks.protection.outlook.com/?url=https%3A%2F%2Fjournals.plos.org%2Fplosone%2Fs%2Ffile%3Fid%3Dba62%2FPLOSOne_formatting_sample_title_authors_affiliations.pdf&data=05%7C02%7Coliver.runswick%40kcl.ac.uk%7Ca38d90e72c0b4149f6a308dcb4ff158c%7C8370cf1416f34c16b83c724071654356%7C0%7C0%7C638584256008996074%7CUnknown%7CTWFpbGZsb3d8eyJWIjoiMC4wLjAwMDAiLCJQIjoiV2luMzIiLCJBTiI6Ik1haWwiLCJXVCI6Mn0%3D%7C0%7C%7C%7C&sdata=VllNLxtQ3rOQ8sd6hG7RHUm688E%2B4DT09gyhELb8zis%3D&reserved=0)

*Response: The manuscript and associated files have been edited to meet the PLOS ONE requirements for style and naming conventions.*

2. Thank you for stating the following financial disclosure:

“OR received contract research funding from FitXR [https://fitxr.com/](https://eur03.safelinks.protection.outlook.com/?url=https%3A%2F%2Ffitxr.com%2F&data=05%7C02%7Coliver.runswick%40kcl.ac.uk%7Ca38d90e72c0b4149f6a308dcb4ff158c%7C8370cf1416f34c16b83c724071654356%7C0%7C0%7C638584256009001357%7CUnknown%7CTWFpbGZsb3d8eyJWIjoiMC4wLjAwMDAiLCJQIjoiV2luMzIiLCJBTiI6Ik1haWwiLCJXVCI6Mn0%3D%7C0%7C%7C%7C&sdata=Kj0xbnDeNfGUpMCAusStLAiDzkd0bUlgf9ffJdsqFxE%3D&reserved=0)”

Please state what role the funders took in the study.  If the funders had no role, please state: "The funders had no role in study design, data collection and analysis, decision to publish, or preparation of the manuscript."

If this statement is not correct you must amend it as needed.

Please include this amended Role of Funder statement in your cover letter; we will change the online submission form on your behalf.

*Response: Thank you, the funders how no role in the research at all. This has been added to the cover letter.*

3. Thank you for stating the following in the Competing Interests section:

“OR received contract research funding from FitXR”

Please confirm that this does not alter your adherence to all PLOS ONE policies on sharing data and materials, by including the following statement: "This does not alter our adherence to  PLOS ONE policies on sharing data and materials.” (as detailed online in our guide for authors [http://journals.plos.org/plosone/s/competing-interests](https://eur03.safelinks.protection.outlook.com/?url=http%3A%2F%2Fjournals.plos.org%2Fplosone%2Fs%2Fcompeting-interests&data=05%7C02%7Coliver.runswick%40kcl.ac.uk%7Ca38d90e72c0b4149f6a308dcb4ff158c%7C8370cf1416f34c16b83c724071654356%7C0%7C0%7C638584256009006449%7CUnknown%7CTWFpbGZsb3d8eyJWIjoiMC4wLjAwMDAiLCJQIjoiV2luMzIiLCJBTiI6Ik1haWwiLCJXVCI6Mn0%3D%7C0%7C%7C%7C&sdata=Y9Nbn4zQhcavm5Ywyge4UVkCoxNgTzisR1P4TCI%2BUlU%3D&reserved=0)).  If there are restrictions on sharing of data and/or materials, please state these. Please note that we cannot proceed with consideration of your article until this information has been declared.

Please include your updated Competing Interests statement in your cover letter; we will change the online submission form on your behalf.

*Response: Thank you for this note, the funders agreed to fully open science in this project and all data and materials can be shared. The suggested statement has been added to the cover letter.*

4. We note that Figure 1 in your submission contain copyrighted images. All PLOS content is published under the Creative Commons Attribution License (CC BY 4.0), which means that the manuscript, images, and Supporting Information files will be freely available online, and any third party is permitted to access, download, copy, distribute, and use these materials in any way, even commercially, with proper attribution. For more information, see our copyright guidelines: [http://journals.plos.org/plosone/s/licenses-and-copyright](https://eur03.safelinks.protection.outlook.com/?url=http%3A%2F%2Fjournals.plos.org%2Fplosone%2Fs%2Flicenses-and-copyright&data=05%7C02%7Coliver.runswick%40kcl.ac.uk%7Ca38d90e72c0b4149f6a308dcb4ff158c%7C8370cf1416f34c16b83c724071654356%7C0%7C0%7C638584256009011636%7CUnknown%7CTWFpbGZsb3d8eyJWIjoiMC4wLjAwMDAiLCJQIjoiV2luMzIiLCJBTiI6Ik1haWwiLCJXVCI6Mn0%3D%7C0%7C%7C%7C&sdata=lkrcqFBbuyzGcQQiUjAo%2BcNXbfCSaZaRRpHHTfFztnA%3D&reserved=0).

We require you to either (1) present written permission from the copyright holder to publish these figures specifically under the CC BY 4.0 license, or (2) remove the figures from your submission:

a. You may seek permission from the original copyright holder of Figure 1 to publish the content specifically under the CC BY 4.0 license.

We recommend that you contact the original copyright holder with the Content Permission Form ([http://journals.plos.org/plosone/s/file?id=7c09/content-permission-form.pdf](https://eur03.safelinks.protection.outlook.com/?url=http%3A%2F%2Fjournals.plos.org%2Fplosone%2Fs%2Ffile%3Fid%3D7c09%2Fcontent-permission-form.pdf&data=05%7C02%7Coliver.runswick%40kcl.ac.uk%7Ca38d90e72c0b4149f6a308dcb4ff158c%7C8370cf1416f34c16b83c724071654356%7C0%7C0%7C638584256009016795%7CUnknown%7CTWFpbGZsb3d8eyJWIjoiMC4wLjAwMDAiLCJQIjoiV2luMzIiLCJBTiI6Ik1haWwiLCJXVCI6Mn0%3D%7C0%7C%7C%7C&sdata=zMuA%2BWWATKvq2fjtcx0gOdE9MEp9uXIa8pcoRM6RMJI%3D&reserved=0)) and the following text:

“I request permission for the open-access journal PLOS ONE to publish XXX under the Creative Commons Attribution License (CCAL) CC BY 4.0 ([http://creativecommons.org/licenses/by/4.0/](https://eur03.safelinks.protection.outlook.com/?url=http%3A%2F%2Fcreativecommons.org%2Flicenses%2Fby%2F4.0%2F&data=05%7C02%7Coliver.runswick%40kcl.ac.uk%7Ca38d90e72c0b4149f6a308dcb4ff158c%7C8370cf1416f34c16b83c724071654356%7C0%7C0%7C638584256009021958%7CUnknown%7CTWFpbGZsb3d8eyJWIjoiMC4wLjAwMDAiLCJQIjoiV2luMzIiLCJBTiI6Ik1haWwiLCJXVCI6Mn0%3D%7C0%7C%7C%7C&sdata=hCOZhOo%2BIaNewEhnEaAuzy0jr7G2p50ubEU45ZYJs18%3D&reserved=0)). Please be aware that this license allows unrestricted use and distribution, even commercially, by third parties. Please reply and provide explicit written permission to publish XXX under a CC BY license and complete the attached form.”

Please upload the completed Content Permission Form or other proof of granted permissions as an "Other" file with your submission.

In the figure caption of the copyrighted figure, please include the following text: “Reprinted from [ref] under a CC BY license, with permission from [name of publisher], original copyright [original copyright year].”

b. If you are unable to obtain permission from the original copyright holder to publish these figures under the CC BY 4.0 license or if the copyright holder’s requirements are incompatible with the CC BY 4.0 license, please either i) remove the figure or ii) supply a replacement figure that complies with the CC BY 4.0 license. Please check copyright information on all replacement figures and update the figure caption with source information. If applicable, please specify in the figure caption text when a figure is similar but not identical to the original image and is therefore for illustrative purposes only.

*Response: We have removed the right-hand side of Figure 1 that included the copyrighted material. All figures now comply with the CC BY 4.0 license. The new figure 1 has been uploaded with the revisions.*

5. Please remove your figures from within your manuscript file, leaving only the individual TIFF/EPS image files, uploaded separately. These will be automatically included in the reviewers’ PDF.

*Response: All figures have been removed from the manuscript.*

**Reviewers' comments:**

**Comments to the Author**

**Reviewer #1:** 

This paper describes a study where investigators aimed to carefully match a 3D commercially available immersive exercise-based application with a 2D (semi-immersive) simulation. The paper is of interest to this reviewer; however, methods require clarification and a more modest interpretation of the results. One concern is the claim that the gaming conditions are matched when in fact there are differences in sensory experience (haptics) and feedback that may explain some of the condition differences beyond level of immersion.

*Response:* *Thank you for the constructive and helpful review of the work. We have endeavoured to clarify the methods and provide a more nuanced interpretation of our results. We have also edited the language in the manuscript to better communicate that it is the visual and auditory stimuli that are matched, and the haptics and feedback are part of what is offered by the more immersive experience. We have added further discussion on the nature of immersion and effects on exercise in the introduction (lines 50-61) and discussion (lines 355-358) including the addition of the recent work of Bird et al. (2024) on the Embodiment–Presence–Interactivity Cube.*

The introduction is well-written framing the study within the context of the importance of activity and the challenges to engage with it regularly. It is useful that the authors reference the COM-B model of behavior change as theoretical anchor for their study. In reviewing the references most of the citations are on healthy young persons and it might be useful to note that in the text.

*Response: Thank you for the positive comment. We have clarified the nature of the populations studied at various points throughout the paper. See for example lines 76-77, 110.*

A few comments on the paragraph that start with line 49-
-Is there a reference for the statement “gaming interfaces as more accessible and affordable (line 52).” Often cost is still cited as a barrier to adoption. In fact, your data indicate that as well.

*Response: This is an important point. We have removed the above statement and edited this paragraph to both better define VR and the perception of and actual cost of VR (lines 48-70).*

-The definition of VR as 3D immersive 3D computer generated simulations (line 53-54) is too restrictive. The suggestion that 2D environments are not VR is limiting. I think there is a difference in immersion of the VR experience. If you want to be restrictive in your definition please provide a citation and rationale. I would suggest you consider immersive compared to semi-immersive VR- rather than VR and no VR.

*Response: Thank you for this interesting point of discussion. We have broadened our discussion in defining VR, included examples, and added some additional literature to support this (Lines 48-70). We have edited the language throughout the manuscript to reflect these definitions and relabelled our conditions as Immersive VR and Screen conditions to reflect this discussion but maintain clarity on the different presentation methods.*

- Can you clarify what you mean by the user can interact simulations that require full body movement (this can be achieved in 2D) and “The element of environmental interaction significantly influences…” what is that is mean by environmental interaction and how is this specific to 3D immersive environments.

*Response: We have also added further information on this section of this element of the definition, interaction involves the user being able to transfer information into the environment using physical movement and cause it to change, this is possible in interactive VR but not video based VR.*

A few comments on the paragraph that starts on line 78
The comments here again might best be framed when studying healthy individuals. There are studies in persons with neurologic health conditions where some of these questions have been addressed using physiologic measures and perception of effort and enjoyment.

*Response: Thank you for this point, we have again added more specific detail on the populations in the studies we have discussed and cited some further work on other clinical populations (lines 93-106, 110).*

-In the literature on VR for persons with stroke there has been work that compared commercial games (Kinect) to custom 2 games with the goal of having equivalence and addressing in this case the limitations of the commercial games.

*Response: Thank you for pointing this out. We were aiming to specifically focusing on the additional benefits of immersive VR that have not been investigated (lines 84-87). We have clarified this and added additional reference to the use of other types of exergames earlier in the work (lines 46-47).*

-There has also been some work comparing the immersive to semi-immersive environment in a cycling simulation for persons with PD.

*Response: Thank you for this point, we have added further citations referring to other populations (lines 102-106), if you are referring to a specific work then please send a citation.*

Method

It is unclear why you have selected RPE as the variable to power the study. It does not appear to be the primary question- Please provide a rationale. Also, the data from Zeng are not similar to yours as they compared their participants in VR and no-VR.

*Response: The primary question of interest is the physical and perceptual responses to immersive VR in the student population we recruited. Based on the previous research discussed we expected that participants would exercise at the same intensities in both conditions, but that they would report lower RPE in more immersive settings. Zeng et al. tested students in conditions that included immersive VR, no-VR and an equivalent screen-based form of semi-immersive VR. This was the best aligned study from which to provide and reasonable effect size estimate. This has been clarified on lines 131-137.*

Was there any age restriction for your participants or was this a sample of convenience and participants had a narrow age range? Please state explicitly under participants.

*Response: There were no limits, but the convenience sample resulted in the narrow range. We have amended to state ‘convenience sample’ in line 136.*

The simulations are not matched. In addition to manipulating the level of immersion the investigators have not controlled for comparable sensory feedback (haptic) and performance feedback. Both factors likely contribute to part of the differences in the group scores. Specifically, as you indicate in the discussion lines 312-318 the haptics have an enhancing effect on presence.

*Response: The aim was to match visual and auditory input and investigate if it is indeed the extra immersion having the effect. We have aimed to clarify this throughout (e.g. lines 121, 338, 400) edited language and this has been added to the limitations section of the Discussion in line 401-402.*

Please describe the four rounds of play and why they were selected or is tis this the default of the game.

*Response:* This was default of the chosen workout in game and is now mentioned in line 159-161. The rounds allow us to see exercise of different intensity within the workout.

Please clarify whether participants returned to their resting HR and RPE during the five-minute rest between conditions. This is important to confirm that participants were at their baseline physiology before each condition. Given that you counter-balanced the order of conditions you may analyze your resting data to confirm that they were equivalent between conditions.

*Response: Participants returned to their resting HR (while standing) between conditions. Thank you for the advice on this, we have included an extra analysis of the resting data to confirm this on line 176-179.*

Please explain why you chose the CR10 Borg instead of the original scale.

*Response: Due to participants needing to report while playing, we found the CR10 more intuitive for them to use. We have added a sentence on line 197-200 to explain*

Tolerability, feasibility and acceptability typically have a priori metrics (eg. Score of X on the SSQ) is there a reason you did not chose to do this? Please notes this as a limitation of the study. It is not clear why some of the data considered qualitative when the responses are yes and no and they are counted. Are these data just descriptive?

*Response: We included these as descriptive measures to capture additional data alongside the primary questions around psychological and physiological response. As far as we are aware there are no standard interpretations in responses to the SSQ, these data were aimed to be an initial description of tolerability in each condition, analysis is reported on lines 322-325. We have noted the lack of a priori metrics in the limitations (Lines 411-412). Thank you for the point on the ‘qualitative’ questions, this was an issue with language use and we have changed this to refer to subjective feedback from participants (lines 208-375). We used positivistic approach to analyse these responses and provide some descriptive data to supplement the other findings.*

The analysis of your VO2 data with 27 for the 30 second epochs is interesting. How did you adjust the Bonneferone correction for that analysis? Your report analyses by round but do not describe that in the methods.

*Response: Thank you for spotting this, we had originally analysed all 30 second epochs but this was very difficult to communicate clearly with post-hoc analysis included, so we instead report analysis of the average of the epochs in each of the four rounds, so we only have four repeated measures. Bonferroni correction is applied to the post-hoc analysis. We have ensured this is clear on lines 222-228.*

It would be better to normalize HR by age rather than present the raw values.

*Response: Thank you for this useful comment, we have converted all the HR results to % of age predicted max. This has been updated in text on lines (247-260) and in Fig 2.*

Was the SSQ administered at baseline and after each condition? You need a baseline assessment for it to be valid. You could analyze it with one way anova with three levels- baseline, semi and full.

*Response: We did not include a baseline because it is not possible to measure ‘simulation sickness’ when no in a simulation. The questions in the SSQ refer specifically to the simulation. The key question is if the more immersive conditions cause any more simulation sickness than the screen-based condition. This analysis is on lines 322-325.*

A bit more explanation of the scoring on the PAAS may help the reader understand your data. Hard to know what a difference between 2.5 and 1.7 means.

*Response: Detail has been added on line 203-205.*

Analysis-
Figure are nice

*Response: Thank you for including the positive feedback!*

Discussion

To interpret the data it seems like you are not using a significant interaction as a measure of an effect and often interpret the main effect of condition as a finding. It would be useful to state that clearly and also to consider whether there is a meaning beyond statistics to the differences. For example, if the HR were normalized one could see if they are exercising at a meaningful intensity. Or with RPE you state that is similar but it is between 10 and 15% lower with the semi VR.

*Response: This is correct, while we report interaction effects to see if the positive effects of the more immersive conditions decrease over time, the effect of interest is the main effect of condition on each variable. We have added detail on the differences in VO2 between conditions (lines 277-278) and* we have also adjusted the HR to be a % age predicted mass to help with this communication of meaningful differences in intensity (see Fig 2.) and lines 259-260.

The conditions not being matched should be addressed as limitation lines 310-315.
Not stating the feasibility and acceptability a priori and measuring the SSQ at baseline are also limitations.

*Response: This as all been acknowledged in the limitations (lines 392-416).*

Finally, not offering feedback in the semi VR is a limitation. Or at the very least along with the haptics speaks to have alternate explanations to immersion as solely causing the findings.

*Response: This is an important point, it is one of the purposes of investigating the immersive VR compared to the screen, but out design cannot determine which elements of the more immersive condition underpin the effects. This has been added to line 298-402.*

Some of your statements should be eliminated or tempered- lines 315-318 are not supported by your data with healthy participants.

*Response: These statements have been removed as part of a more nuanced discussion of the results and implications.*

Suggested specific revisions
Abstract
Line 7 Exactly matched- revised to closely matched

*Response: this has been amended and clarified on lines 5 and 6.*

**Reviewer #2:**

Introduction (Lines 25-98):
1. Detailed Mechanisms:
o Line 32-34: The introduction could benefit from a more detailed discussion on the specific mechanisms by which VR might influence physiological and psychological outcomes during exercise. This will help in setting a clear context for the study.

*Response: We have expanded the introduction in several ways, including discussion of possible mechanisms (e.g. lines 63-67) and more depth in the discussion of previous literature to set a clear context for the work (lines 93-106).*

2. Potential Negative Effects of VR:
o Line 70-72: The introduction should include a discussion on the potential negative effects of VR exercise, such as dizziness and nausea. This is important for providing a balanced view of the technology's potential.

*Response: Thank you for this point, it is not something that we have found many people actually experience, but the potential for this is important to note and has been included in the introduction alongside other issues such as perceived (but not actual) cost. Lines 67-70:104-106) .*

Methodology (Lines 99-141):
1. Sample Size Justification:
o Line 99-101: The sample size (n=17) is quite small. Power analysis details should be provided, explaining why this sample size was chosen and how it is sufficient to detect meaningful differences.

*Response: The primary question of interest is the physical and perceptual responses to immersive VR in the student population we recruited. Based on the previous research discussed we expected that participants would exercise at the same intensities in both conditions, but that they would report lower RPE in more immersive settings. Zeng et al. tested students in conditions that included immersive VR, no-VR and an equivalent screen-based form of semi-immersive VR. This was the best aligned study from which to provide and reasonable effect size estimate. This has been clarified on lines 131-136.*

2. Participant VR Experience:
o Line 99-101: Information about participants' prior experience with VR should be included. VR's novelty could significantly influence the results, and this potential confounder should be acknowledged and controlled if possible.

*Response: Participants were required to be novices to VR. This has been added to lines 138-139. We have also expanded on the novelty issue in the discussion on lines 390-391: 421-423.*

3. Inclusion and Exclusion Criteria:
o Line 110-112: The criteria for including and excluding participants should be more detailed, including specific medical conditions that would disqualify participants and any other relevant factors.

*Response: Specific medical conditions and inclusion and exclusion criteria are listed on lines 138-142, otherwise there were no further specific exclusion criteria. These criteria were included in recruitment documentation, so no participants volunteered and were subsequently excluded. The exact anthropometric details are also listed here.*

4. Participant Familiarization:
o Lines 99-101: It is unclear how participants were familiarized with the VR equipment before the study. Providing details on this process is crucial for understanding the participants' comfort and proficiency with the VR system.

*Response: Prior to use of the headset, participants received verbal instructions for controller use and navigating the virtual and physical environment safely. Participants were then required to navigate the menus themselves to show proficiency in the use of the headset and app. Once in the app participants navigated to the workout themselves. This has been included in lines 150-153*

5. Control for Novelty Effect:
o Lines 105-108: The study should address whether any control measures were taken to account for the novelty effect of using VR, as this can significantly impact participants' responses.

*Response: This is an important point and an issue in much of the literature in this area. This is one of the reasons that we recorded physiological responses every 30 seconds over the course of the workout, to establish if effects are prominent early in the workout. This has been noted on lines 193-194. We did not see any interaction effects where effects of the VR workout decreased over time. However, we did not conduct multiple visits to the lab to establish if effects were consistent across longer time periods. We have addressed this in the limitations section on lines 398-399.*

Results (Lines 206-270):
1. Effect Sizes:
o Line 206-215: Effect sizes should be reported alongside p-values for all statistical tests to provide a clearer picture of the practical significance of the findings.

*Response: All reported p values have either partial eta squared or cohen’s d reported.*

2. Heart Rate Discrepancy:
o Line 207-215: The heart rate results show no significant difference between VR and non-VR conditions, yet there is a main effect of time. The authors should explore and discuss possible reasons for this discrepancy.

*Response: This is not a discrepancy. The main effect of time is simply showing that heart rate increases over time during exercise and as exercise intensity changes across the rounds. This is a common physiological mechanism.*

3. Post-Hoc Analyses:
o Line 221-227: Conduct and report post-hoc analyses for VO2 and heart rate data to clarify where specific differences lie between the VR and non-VR conditions.

*Response: Full post hoc analysis has been added with appropriate effect size calculations see lines 247-260: 272-278: 289-294.*

4. Perceived Exertion:
o Line 232-236: The perceived exertion results are higher for the VR condition, contrary to some previous findings. The discussion should include potential reasons for this difference, such as the impact of the immersive experience or differences in individual perception.

*Response: We have including discussion on the fact that we allowed participants to choose their exercise intensity, the majority of other work that has found the reduced RPE as add some kind of control of workload rather than allowing natural behaviour. This was addressed in the discussion in line 348-358.*

Discussion (Lines 284-362):
1. COM-B Model Discussion:
o Line 326-338: The discussion around the COM-B model appears speculative. This section should be more closely tied to the actual data from the study, providing concrete examples of how the study's findings support or challenge the COM-B model.

*Response: This is an important point, we have aimed to be more nuanced in our language and included some examples of how our data links to these point (lines 379-391).*

2. Novelty Effect:
o Line 343-344: The discussion should consider the potential impact of the novelty effect on the results and suggest ways to mitigate this in future studies.

*Response: This is mentioned in line 393-400– ‘*Participants were new to VR, and many reported they took part due to the novelty and were interested in trying VR (Table 2). This may lead to higher levels of interest and effort in the VR condition and future research should investigate the use of VR over longer training periods.’

3. Comparison with Previous Studies:
o Line 350-352: A more detailed comparison with previous studies that found lower perceived exertion in VR conditions is needed, exploring why the current study found different results.

*Response: Response: We have including discussion on the fact that we allowed participants to choose their exercise intensity, the majority of other work that has found the reduced RPE as add some kind of control of workload rather than allowing natural behaviour. This was addressed in the discussion in line 355-358.*

4. Long-term Adherence:
o Line 359-360: The implications of the findings for long-term adherence to VR exercise programs should be discussed, considering the novelty might wear off over time.

*Response: This has been addressed in the discussion and limitations on lines 390-398 and 421-423.*

5. Limitations Section:
o Add a dedicated "Limitations" subsection to discuss limitations such as the small sample size, potential novelty effects, and any other relevant factors.

*Response: This can be found on line 392-416.*

Minor Revisions:
Abstract (Lines 1-19):
1. Limitations and Future Directions:
o Line 19: Include a sentence on the study's limitations and potential directions for future research to provide a more balanced overview of the study's contributions and areas for improvement.

*Response: Limitations have been acknowledged and a sentence has been added at the end of abstract – lines 16-18.*

2. Minor Grammatical Errors:
o Line 13: Minor grammatical errors and typos should be corrected for clarity.

*Response: This has been reworded, now on lines 13-16.*

Introduction:1. References:
o Ensure all references are up-to-date and relevant. Some references, particularly those discussing the benefits of VR in exercise, could be expanded.

*Response: We have expanded upon study by Zeng et al and Mologne et al and have included clinical population studies (lines 84-106). We have also added recent work from Bird et al 2024 and Teixeira et al 2024 (lines 84-87: 101-103) that support the rationale.*

Methodology (Lines 99-141):
1. Participant Recruitment and Criteria:
o Line 105-108: Provide more detail on participant recruitment and the inclusion/exclusion criteria to enhance the study's transparency and reproducibility.

*Response: This has been added on lines 136-142.*

2. Experimental Setup Diagram:
o Line 127: Include a diagram of the experimental setup showing the VR and non-VR conditions to help readers visualize the experimental environment.

*Response: We have attempted to produce this, but the diagram simply consists of a person in a room space with or without a head mounted display. We did not feel this added anything further to Fig1 that display the experimental set up for the immersive VR condition. This screen condition is identical but without the HMD. We have noted this on lines 166-167.*

3. RPE Reporting in VR Condition:
o Line 154-159: Clarify how participants reported RPE in the VR condition when they could not see the scale. Describe any training or familiarization they received for this task.

*Response: This has been clarified in line 197-200 and noted in the limitations on line 408-411.*

Results (Lines 206-270):
1. Error Bars in Figures:
o Line 207-215: Add error bars to Figures 2 and 3 to help readers assess the variability in the data.

*Response: Both these figures already contained error bars. We have added description of the error bars to the figure captions to ensure that this was clear.*

Discussion:
1. Minor Grammatical Corrections:
o Line 352: Minor grammatical corrections are needed for clarity.

*Response: This has been reworded and some detail added based on other comments.*

References:1. Formatting and Completeness:
o Line 364: Verify that all references are correctly formatted and complete, including checking DOIs and ensuring all cited works are listed in the reference section.

*Response: All the references have been checked, edited, and formatted.*
