# Supplementary material for: Acute psychological and physiological benefits of exercising with virtual reality
Source: PLoS One. 2024 Dec 18;19(12):e0314331. doi: 10.1371/journal.pone.0314331 (PMC11654962; doi:10.1371/journal.pone.0314331)
Supplement: Attachment — Submitted filename: Response to Reviewers.docx [file pone.0314331.s003.docx]

Response: Thank you to the editors and reviewers for the support in improving this paper. We have fully addressed all the additional comments below. Responses can be found under each of the reviewer comments and additions to the manuscript in red text.

Review Comments to the Author

Reviewer #2: The authors have successfully addressed my previous critiques and made the necessary revisions. The explanations regarding the statistical methods and participant selection have been clarified, and the inclusion of post-VR participant feedback has added value to the study. These revisions have strengthened the methodological rigor and reinforced the validity of the results.

Response: Thank you for your positive comments and your help in improving the manuscript.

Reviewer #3: Thank you for the opportunity to review this manuscript. The study addresses a highly relevant topic, investigating the psychological and physiological effects of exercise in virtual reality (VR), a field of growing interest. The use of commercially available VR devices makes the findings relevant to the general public. Among the strengths of the study, the rigorous comparison between VR exercise and a 2D screen equivalent stands out, with statistically significant results showing improvements in participants' psychological and physiological responses during the VR condition.

Response: Thank you for your positive comments and your help in improving the manuscript.

However, there are several sections that require adjustments to improve the clarity and coherence of the article:

Introduction: This section could be streamlined to avoid repetition of general concepts about the benefits of exercise, which are already well known. I suggest focusing more on the unique contribution of this study, namely the direct comparison between VR and 2D screen-based exercise with acute physiological and psychological measurements. This will highlight the research gap the study aims to address, avoiding unnecessary details on established knowledge.

Response: Thank you for this comment, we have reworked the introduction to enhance brevity and more clearly get to the main aims for the study. For example, we have cut much of the repetition from the first paragraph and included new text to direct the reader straight to the point of this work (now Lines 24-33). We have synthesised discussion of barriers in the second paragraph (now Lines 34-44) and synthesised the fourth and fifth paragraphs that discuss previous work on VR for exercise (e.g. line 72-74; 80-85). Overall, we reduced the introduction by over 200 words and streamlined the narrative to focus more on the unique contribution here (see lines 41; 47; 64-66).

Methodology: The description of the experimental protocol needs more clarity. It is important to specify: How the order of conditions (VR and non-VR) was counterbalanced to avoid potential sequence effects that could influence participants' performance and psychological responses.

Response: Thank you for this comment, we had already noted that conditions were counterbalanced (now on line 157). As this study only has two conditions participants were simply allocated alternation orders.

The exact duration of the rest period between experimental sessions should be more precisely indicated to ensure that participants adequately recovered between trials, reducing the risk of cumulative fatigue that might distort the results.

Response: We have given an approximate value of 5 minutes (line 159) because the rest period was governed by participants returning to resting heart rate. We have added this detail (line 159-160) for clarity.

Data Analysis: A more detailed explanation of how violations of sphericity in the ANOVA were handled would be useful, along with information on any statistical adjustments made (e.g., Greenhouse-Geisser or Huynh-Feldt corrections). This would improve the transparency of the analysis and strengthen the robustness of the results.

Response: This is outlined in the data analysis on, now on lines 212-214.

‘Violations of sphericity were corrected by adjusting the degrees of freedom using the Greenhouse–Geisser correction when ε was less than 0.75 and the Huynh–Feldt correction when greater than 0.75.’

Limitations of the study: The discussion of limitations should be expanded. The novelty of VR technology may have influenced the results, as many participants were new to this experience and may have reported higher levels of enjoyment and motivation compared to a more familiar device. This point should be mentioned, and it would be appropriate to suggest longitudinal studies to assess whether these effects persist over time once the novelty effect wears off.

Response: The novelty points and longitudinal research is outlines on line 378-383.

‘Firstly, novelty may play a role in the findings reported here from acute bouts of exercise. Participants were new to VR, and many reported they took part due to the novelty and were interested in trying VR (Table 2). This may lead to higher levels of enjoyment and effort in the VR condition and f uture research should investigate the use of VR over longer training periods, considering how these effects compare over time with diminishing novelty.’

Generalizability of the results: It is important to better address the fact that the participant sample consisted of young, physically active individuals, which limits the generalizability of the findings to other populations, such as older or less active individuals. I suggest including a discussion on how results might differ in these groups and proposing future research to explore these variables in different cohorts.

Response: We had already mentioned this (lines 388-390) but have expanded the point further.

‘Additionally, our cohort was relatively young and active. Therefore, our findings may not be applicable to the wider general population or older aged adults. Future research should focus on these populations, where it is possible positive impacts could be greater due lower baseline levels fitness.’

Conclusion section: The conclusions should be more cautious. While the results are promising, it is important not to overgeneralize. Specifically, it should be emphasized that the observed benefits may be specific to the young and healthy sample studied, and that further research is needed to confirm whether these advantages apply to other populations.

Response: Thank you for this point. We have reworded the conclusion on lines 404-410.
