# Supplementary material for: Acute psychological and physiological benefits of exercising with virtual reality
Source: PLoS One. 2024 Dec 18;19(12):e0314331. doi: 10.1371/journal.pone.0314331 (PMC11654962; doi:10.1371/journal.pone.0314331)
Supplement: Attachment — Submitted filename: Reviewer Comments.pdf [file pone.0314331.s001.pdf]

Peer Review for Manuscript PONE-D-24-05252: "Acute psychological and physiological benefits of exercising with virtual reality"

Major Revisions:

Introduction (Lines 25-98):

1. **Detailed Mechanisms:**
   - **Line 32-34:** The introduction could benefit from a more detailed discussion on the specific mechanisms by which VR might influence physiological and psychological outcomes during exercise. This will help in setting a clear context for the study.
2. **Potential Negative Effects of VR:**
   - **Line 70-72:** The introduction should include a discussion on the potential negative effects of VR exercise, such as dizziness and nausea. This is important for providing a balanced view of the technology's potential.

Methodology (Lines 99-141):

1. **Sample Size Justification:**
   - **Line 99-101:** The sample size (n=17) is quite small. Power analysis details should be provided, explaining why this sample size was chosen and how it is sufficient to detect meaningful differences.
2. **Participant VR Experience:**
   - **Line 99-101:** Information about participants' prior experience with VR should be included. VR's novelty could significantly influence the results, and this potential confounder should be acknowledged and controlled if possible.
3. **Inclusion and Exclusion Criteria:**
   - **Line 110-112:** The criteria for including and excluding participants should be more detailed, including specific medical conditions that would disqualify participants and any other relevant factors.
4. **Participant Familiarization:**
   - **Lines 99-101:** It is unclear how participants were familiarized with the VR equipment before the study. Providing details on this process is crucial for understanding the participants' comfort and proficiency with the VR system.
5. **Control for Novelty Effect:**
   - **Lines 105-108:** The study should address whether any control measures were taken to account for the novelty effect of using VR, as this can significantly impact participants' responses.

Results (Lines 206-270):

1. **Effect Sizes:**
   - **Line 206-215:** Effect sizes should be reported alongside p-values for all statistical tests to provide a clearer picture of the practical significance of the findings.

2. **Heart Rate Discrepancy:**
    - **Line 207-215:** The heart rate results show no significant difference between VR and non-VR conditions, yet there is a main effect of time. The authors should explore and discuss possible reasons for this discrepancy.
3. **Post-Hoc Analyses:**
    - **Line 221-227:** Conduct and report post-hoc analyses for VO2 and heart rate data to clarify where specific differences lie between the VR and non-VR conditions.
4. **Perceived Exertion:**
    - **Line 232-236:** The perceived exertion results are higher for the VR condition, contrary to some previous findings. The discussion should include potential reasons for this difference, such as the impact of the immersive experience or differences in individual perception.

Discussion (Lines 284-362):

1. **COM-B Model Discussion:**
    - **Line 326-338:** The discussion around the COM-B model appears speculative. This section should be more closely tied to the actual data from the study, providing concrete examples of how the study's findings support or challenge the COM-B model.
2. **Novelty Effect:**
    - **Line 343-344:** The discussion should consider the potential impact of the novelty effect on the results and suggest ways to mitigate this in future studies.
3. **Comparison with Previous Studies:**
    - **Line 350-352:** A more detailed comparison with previous studies that found lower perceived exertion in VR conditions is needed, exploring why the current study found different results.
4. **Long-term Adherence:**
    - **Line 359-360:** The implications of the findings for long-term adherence to VR exercise programs should be discussed, considering the novelty might wear off over time.
5. **Limitations Section:**
    - Add a dedicated "Limitations" subsection to discuss limitations such as the small sample size, potential novelty effects, and any other relevant factors.

Minor Revisions:

Abstract (Lines 1-19):

1. **Limitations and Future Directions:**

- **Line 19:** Include a sentence on the study's limitations and potential directions for future research to provide a more balanced overview of the study's contributions and areas for improvement.

2. **Minor Grammatical Errors:**
    - **Line 13:** Minor grammatical errors and typos should be corrected for clarity.

Introduction:

1. **References:**
    - Ensure all references are up-to-date and relevant. Some references, particularly those discussing the benefits of VR in exercise, could be expanded.

Methodology (Lines 99-141):

1. **Participant Recruitment and Criteria:**
    - **Line 105-108:** Provide more detail on participant recruitment and the inclusion/exclusion criteria to enhance the study's transparency and reproducibility.
2. **Experimental Setup Diagram:**
    - **Line 127:** Include a diagram of the experimental setup showing the VR and non-VR conditions to help readers visualize the experimental environment.
3. **RPE Reporting in VR Condition:**
    - **Line 154-159:** Clarify how participants reported RPE in the VR condition when they could not see the scale. Describe any training or familiarization they received for this task.

Results (Lines 206-270):

1. **Error Bars in Figures:**
    - **Line 207-215:** Add error bars to Figures 2 and 3 to help readers assess the variability in the data.

Discussion:

1. **Minor Grammatical Corrections:**
    - **Line 352:** Minor grammatical corrections are needed for clarity.

References:

1. **Formatting and Completeness:**
    - **Line 364:** Verify that all references are correctly formatted and complete, including checking DOIs and ensuring all cited works are listed in the reference section.
